# Undergraduate medical education amidst volatility, uncertainty, complexity, ambiguity

**DOI:** 10.3389/fmed.2025.1666718

**Published:** 2025-10-28

**Authors:** Troy Camarata, Angelina Spizzieri, Anand Kulkarni

**Affiliations:** Baptist University College of Osteopathic Medicine, Memphis, TN, United States

**Keywords:** medical education, artificial intelligence, VUCA, education technology, faculty retention

## Abstract

Undergraduate medical education, especially in the United States, is being shaped by an environment that is changing at an unprecedented pace, creating new challenges for institutions, faculty, and students. For medical education to adapt to the changing environment, influencing factors must be identified and categorized, allowing for the development of strategies that work within the new landscape. Volatility, uncertainty, complexity, and ambiguity (VUCA) is a method to analyze the factors driving change. Here, we apply the VUCA framework to undergraduate medical education, highlighting key influencing factors including artificial intelligence, decentralized approaches to learning, expansion of medical schools, and new demands on faculty and students. The goal is to identify how these influences are impacting medical education allowing for creative and innovative strategies to be employed that mitigate disruptive forces and take advantage of new opportunities to the benefit all stakeholders.

## Introduction

Undergraduate medical education (UME) in the United States is undergoing significant transformation, shaped by the interplay of multiple interactive factors. These changes are driving an environment characterized by volatility, uncertainty, complexity, and ambiguity (VUCA) that poses both challenges and opportunities for educators and institutions. Originally coined in 1987 by the U.S. Army War College to describe the turbulent post-Cold War landscape, volatility, uncertainty, complexity, and ambiguity (VUCA) has been applied to multiple areas of business, human resources, and even healthcare (1–5). A conceptual framework for the components of VUCA has been reviewed elsewhere but is briefly summarized here (2, 4). Volatility refers to the speed of change in an industry or market and describes the intensity of fluctuations over time. Uncertainty or unpredictability refers to the extent to which one can logically predict the future with confidence. The more uncertain the world is, the harder it is to predict. Complexity refers to the number of factors that need to be considered. The more numerous, varied, and interconnected the factors, the more complex the environment. Lastly, ambiguity refers to a lack of clarity about how to interpret a situation due to incomplete information or conflicting evidence.

The practice of medicine, which encompasses undergraduate and graduate education, regulation, ethics, public perception, along with many other domains, is an incredibly complex and overwhelming topic. To focus, the VUCA framework is applied to UME. We outline four primary drivers directly impacting medical education transformation, of which institutions can respond and retain some measure of control. These drivers include integration of artificial intelligence (AI), increased demand for faculty driven by the proliferation of new medical schools, the rise of technology-based, interactive, and technology driven educational resources, and shifting generational expectations, which further exacerbates the VUCA landscape (6, 7). It is imperative for institutions and educators to recognize emerging challenges and adapt to the evolving landscape of UME. This article analyzes the shifting environmental factors associated with VUCA and anticipates their cumulative impact on the landscape of medical education providing a framework for institutions, faculty, and students to adapt to this changing environment.

## Artificial intelligence in medical education

Artificial intelligence (AI) is revolutionizing medical education by introducing innovative tools that enhance learning, assessment, and clinical training. AI-driven platforms, such as intelligent tutoring systems and diagnostic tools, offer personalized learning experiences and data-driven insights into student performance (8). However, this rapid technological advancement has created a volatile environment. All involved members of medical education must quickly adapt to these tools, which are often expensive and require substantial training to implement effectively. Questions regarding training for both faculty and students have been raised, but without a clear understanding of how and when this training will occur (9, 10).

The integration of AI brings uncertainty (Table 1). While AI holds immense promise in improving learning outcomes, its long-term impact on critical thinking, diagnostic reasoning, and professional judgment remains unclear (11). Use of AI in medicine to protect patient safety and privacy along with how to train students and residents in these areas remains unclear (12). Additionally, there are ethical concerns regarding AI biases and unequal access to AI-powered resources, potentially widening the gap between well-funded and under-resourced institutions (13). Interestingly, it is possible for AI to level the playing field for countries that are not normally included in research enterprises. One scoping review found that nearly one-third of published medical education research using AI originated from countries outside of North America and Europe (14). The authors suggest the less resource intensive AI platforms can allow for more inclusive research practices and outputs.

**Table 1:** Summary of volatility, uncertainty, complexity, and ambiguity in undergraduate medical education.

Definition	Introduction of AI in medical education	Increasing demand for faculty due to new medical schools	Interactive, modern educational resources	Shifting expectations from new generations
Volatility	Rapid technological advancements and AI integration demand swift adaptation by faculty and students	Increase in new medical schools and heightened competition for limited faculty and resources	Rapid evolution of learning technologies creates constant pressure to update teaching methods	Escalating educator workload driven by evolving and dynamic student expectations
The speed of change in an industry or market and the intensity of fluctuations over time				
Uncertainty	Unpredictable long-term impact of AI on reasoning, privacy, ethics, and bias	Unstable outlook for faculty compensation, retention, and sustainability	Uncertainty about the selection, implementation, and effectiveness of educational technologies	Unclear consequences of changing generational values on educational goals and outcomes
The extent to which the future can be predicted with confidence				
Complexity	Challenges in integrating AI into curricula while navigating privacy, training needs, and evidence-based approaches	Complex faculty management involving workload, growth, retention, and governance amid institutional expansion	Complex decisions around implementing, accessing, and evaluating educational technologies in a crowded and sometimes unreliable market	Tension between maintaining foundational educational principles and meeting demands for interactive, digital, and flexible formats from new-generation learners
The number of interconnected variables that must be considered				
Ambiguity	Ambiguity surrounding AI competencies, engagement strategies, and its future role in clinical practice	Lack of clear guidelines on faculty retention and managing politicized or sensitive curricular content	Unclear best practices for guiding digital tool use while mitigating distractions and negative impacts	Ambiguity in balancing pedagogical innovation with core principles in response to evolving student expectations
Lack of clarity due to incomplete or contradictory information				

The complexity of implementing AI in medical education is significant (Table 1). Educators must navigate the challenges of aligning AI tools with curricular goals while addressing concerns about data privacy and security. Medical education is still in the middle of a shift to active learning strategies and reducing barriers for faculty to develop appropriate activities (15, 16). Adding another significant tool to the classroom and other educational settings will certainly add to the difficulty faculty face with educating students. Attempts to incorporate AI driven tools with other evidence-based learning strategies are underway (17, 18). Despite these hurdles, AI has the potential to reshape medical education by fostering adaptive, efficient, and student-centered learning environments.

Currently, there is a great deal of ambiguity surrounding the long-term impact of AI on medical education and clinical practice. Potential changes in competencies in healthcare, and as a result of health education, have been suggested to match the incorporation of AI usage (19, 20). Several perspectives and reviews have suggested frameworks for the adoption of AI in health education (19, 21). Developing AI literacy amongst students and faculty is becoming clear (22, 23). However, some studies have found conflicting evidence between the perception of AI usage by students and some aspects of creative thinking, emotional engagement, and alterations in brain activity (24, 25). Much remains to be determined regarding AI-induced shifts in medical education, the role of faculty, and new competencies in health practice.

## New medical schools

The proliferation of medical schools in the U.S. has occurred in bursts. Following a nearly three-decade long stagnation, the country is currently experiencing another prolonged burst in new medical schools (26, 66). The current push for both increased class size and creation of new medical schools has been precipitated by analysis suggesting significant physician shortages in the next two decades (27). This has created a volatile environment in medical education, with medical education institutions competing for qualified faculty, limited resources, and clinical training sites.

Expansion of UME comes with great complexity (Table 1). There are significant challenges new or expanding medical schools face, including maintaining student support services, financial flexibility and availability, and logistical issues, both expected and unexpected (28, 66). Although continued action at both state and federal levels is necessary to ensure balanced growth of UME, political shifts can pose significant challenges (29).

The proliferation of medical schools also brings ambiguity, as maintaining consistent educational quality across new and existing schools becomes a pressing concern. Multiple curricular designs are implemented in medical schools from discipline-based, to organ system-based to patient-presentation-based (30). Furthermore, multiple grading schemes are used (pass/fail, letter grade, numerical score) and the length of pre-clerkship curriculum varies from 12 to 24 months (31). Support in the literature for different curriculum types can be easily found along with tales of caution (32–36). The Association for American Medical Colleges (AAMC) and American Association of Colleges of Osteopathic Medicine (AACOM) have created a set of joint competencies to help guide UME (37). How competencies are achieved within the four-to-six-year window allowed by accrediting bodies is up to individual institutions, which may contribute to some level of ambiguity about how to best prepare future medical graduates (38).

Curricular design variability in UME, ranging from discipline-based to systems-based to presentation-based models, diverse grading schemas, and variable pre-clerkship lengths, directly drives faculty demand. Each redesign cycle requires additional educator time for blueprinting, content integration across disciplines, assessment development and standard setting, program evaluation, and accreditation documentation. Newer schools must build these functions from the ground up; expanding schools must run redesign and delivery in parallel. In both cases, the faculty hours, breadth of expertise, and instructional design capacity needed to sustain high-quality programs increase substantially, tightening already competitive faculty markets and intensifying recruitment and retention pressures.

The establishment of new medical schools and the increasing reliance on advanced educational technologies have amplified the demand for skilled educators. However, the supply of qualified faculty remains deficient, creating a volatile staffing environment (67). Many institutions struggle to recruit and retain faculty with the expertise needed to teach specialized subjects, such as anatomy, pathology, and clinical skills. Surveys of medical school faculty reveal a significant level of dissatisfaction. One study which surveyed faculty from 26 medical schools found over 20% strongly considered leaving their position citing poor institutional culture, disengagement and lack of institutional support and self-efficacy (39). Similarly, data from a single institution found 42% of medical school faculty considered leaving citing issues with career progression, work-life balance, and participation in institutional governance (40). The availability of tenure and tenure-track positions is also decreasing, especially for clinical faculty (41). The reduction in tenure and the academic freedom it typically affords may put faculty in tenuous positions with changing state and federal policy changes related to clinically related topics involving reproductive care, social determinants of health, and gender identity (42, 43). National data supports difficulties in faculty retention as the attrition rates reached their highest levels in 2014, the most recent date for available data, since 1985 (67). Much of the faculty data comes from established institutions, yet new medical schools are not immune. A survey of founding faculty from a new medical school found positives such as higher rank, high collegiality, compensation, and professional growth (44). However, challenges were present such as poor opportunities for promotion, reduced scholarly output, and unsatisfactory support from administration. Faculty challenges extend beyond UME. Clinical faculty involved in graduate medical education are also being required to adapt to evolving roles, shifting expectations, and increasingly complex learning environments (45).

Uncertainty about compensation and career opportunities further complicates the issue (Table 1). The opening of numerous new medical schools may lead to increased competition for educators. Such a trend exacerbates faculty shortages and challenges the sustainability of high-quality medical education. The complexity of addressing these shortages involves balancing workload distribution, enhancing professional development opportunities, and creating incentives to retain experienced educators. Some level of ambiguity exists as limited guidelines have been published to help in retaining faculty, although the limited information that has been presented may not be widely instituted (46).

## Interactive and modern educational resources

The transition from passive teaching methods, such as traditional lectures, to interactive and technology-enhanced learning resources is reshaping the delivery of medical education. Tools such as virtual reality (VR), augmented reality (AR), and simulation-based learning offer immersive experiences that enhance knowledge retention and clinical skills (47–49). However, the fast-paced evolution of these tools introduces volatility, as educators struggle to keep up with emerging technologies. Quality control, training, and ensuring the promotion of learning have not kept pace (50).

Institutions face uncertainty about which technologies to adopt and how to integrate them into existing curricula. The COVID-19 pandemic greatly advanced the transition to digital resources and platforms in medical education. These resources can be used to augment traditional curricula (47). Challenges remain in how to ensure ethical standards of implementing digital and other technologies while maintaining personal training in compassion and empathy which are vital to the practice of medicine (51).

The complexity of implementing these tools lies in ensuring equitable access and training for both students and faculty (Table 1). Implementing educational technology that promotes deep learning and knowledge acquisition rather than mere information retrieval remains a significant challenge (52). Previously, knowledge was curated through textbooks, peer-reviewed journals, and by expert educators. With the rise of internet and social media resources, many of the guardrails have been removed and the learner is required to determine the reliability of information. Medical school faculty may now be faced with a new complex challenge of either curating the vast amount of material available through the internet or social media platforms or find novels ways to train students into becoming experts in identifying trustworthy resources (52). The expert opinions of faculty may still not be sufficient. One study found that online videos explaining atrial fibrillation that were created by experts were not nearly as popular as those from non-experts (53). Pitfalls in emerging technologies and resources include the possibility of misinformation which inherently makes the role of both faculty and students much more difficult (47). Despite these challenges, modern educational resources have the potential to revolutionize medical training by making it more engaging, flexible, and relevant to the demands of contemporary healthcare.

Emerging and ever evolving technologies create new levels of ambiguity about how to implement these effectively and to reduce unwanted outcomes. Digital resources provide a great deal of flexibility where the learner can control how and when information is provided (47). Use of resources may also allow for the identification of knowledge gaps; however, negative correlations have been found with academic performance and the number of outside resources students utilize (54, 55). Yet, medical students show a preference for outside resources for learning (56). Use and preference of learning resources by medical students may be at times in conflict with what is best for learning. Potential conflicts are not restricted to learning and cognition but may also be physical. Studies of learning during the COVID-19 pandemic highlighted the increase in computer vision syndrome in medical students (57, 58). Technology-based learning resources are becoming an important part of medical education. Clear guidelines for faculty and institutions that allow for discrimination of trusted sources that promote both learning and physical wellness remain to be studied.

## Shifting expectations from new generations

Looking ahead, medical educators are anticipating a significant shift in methodologies due to the evolving expectations of Gen Z (born between 1995 and 2012) and newer generations of students. Gen Z learners grew up with technology being completely ubiquitous (59). These learners prioritize digital, interactive, and flexible learning environments that align with their tech-savvy and collaborative nature (7). However, the literature suggests that several supportive measures are needed for Gen Z learners to support life skills that are not as prominent compared to older generations (6, 7, 60, 61). This includes skills such as balancing multiple tasks simultaneously, time management, and reading long-form text effectively (61). The inclusion of learner training to bridge gaps in life skills along with the increasing amount of medical knowledge needed in medicine creates a great deal of complexity (Table 1). How to balance theoretical knowledge acquisition with a focus on critical thinking and evaluation, especially in a world where AI usage will increase, will be an ongoing challenge (62).

While the expectations of Gen Z and future generations will drive innovation, they also increase the workload for educators, who must create relevant educational materials, integrate new technologies, be trained and train learners on new technologies, and stay current with the exponential pace of medical knowledge. This increased workload carries the risk of negatively impacting student learning, retention, and satisfaction.

The ambiguity surrounding the long-term implications of these shifts highlights the need for a balanced approach that leverages technology without compromising foundational learning principles. Institutions must invest in faculty support systems and sustainable teaching practices to navigate this changing landscape effectively.

## Conclusion

The emergence of VUCA in medical education underscores the need for adaptability, collaboration, and innovation. By addressing the challenges posed by AI, proliferation of medical schools, modern technology-based educational resources, faculty shortages, and shifting generational expectations, educators and institutions can create a resilient and future-ready medical education system. Although written with the current perspective of medical education in the United States, many of the described VUCA challenges are highly relevant globally. Medical education systems around the world are dealing with similar challenges. For example, faculty shortages and high faculty attrition has been documented in India (63) while challenges in e-learning training of both students and faculty has been investigated in Uganda (64). However, regions and countries have unique challenges that cannot be addressed here. War torn Ukraine is facing extreme challenges with medical education that very much fits the VUCA framework (65). While many additional forces shape medical education, the intention here is to analyze a focused subset with outsized near-term impact through a VUCA lens. We encourage future work to extend this framework to workforce distribution and clinical-system complexity, which merit dedicated treatment. Navigating this VUCA environment requires a commitment to continuous learning, strategic planning, and a shared vision for advancing the quality and accessibility of medical education.

## Data Availability

The original contributions presented in the study are included in the article/supplementary material, further inquiries can be directed to the corresponding authors.
